# CFI: a VR motor rehabilitation serious game design framework integrating rehabilitation function and game design principles with an upper limb case

**DOI:** 10.1186/s12984-024-01373-2

**Published:** 2024-07-01

**Authors:** Chengjie Zhang, Suiran Yu, Jiancheng Ji

**Affiliations:** 1 School of Mechanical Engineering, Shanghai Jiao Tong University, Shanghai, China; 2 Shenzhen Polytechnic University, Shenzhen, China

**Keywords:** VR rehabilitation, Rehabilitation, Game design, Games, Stroke

## Abstract

Virtual reality (VR) Rehabilitation holds the potential to address the challenge that patients feel bored and give up long-term rehabilitation training. Despite the introduction of gaming elements by some researchers in rehabilitation training to enhance engagement, there remains a notable lack of in-depth research on VR rehabilitation serious game design methods, particularly the absence of a concrete design framework for VR rehabilitation serious games. Hence, we introduce the Clinical-Function-Interesting (CFI): a VR rehabilitation serious game design framework, harmonizing rehabilitation function and game design theories. The framework initiates with clinic information, defining game functions through the functional decomposition of rehabilitation training. Subsequently, it integrates gaming elements identified through the analysis and comparison of related literature to provide enduring support for long-term training. Furthermore, VR side-effect and enhancement are considered. Building upon this design framework, we have developed an upper limb VR rehabilitation serious game tailored for mild to moderate stroke patients and aligned our framework with another developed VR rehabilitation serious game to validate its practical feasibility. Overall, the proposed design framework offers a systematic VR rehabilitation serious game design methodology for the VR rehabilitation field, assisting developers in more accurately designing VR rehabilitation serious games that are tailored to specific rehabilitation goals.

## Introduction

Motor disorders are disorders of the nervous system that cause abnormal and involuntary movements [1]. The World Health Organization (WHO) reports that over one billion individuals are affected by these disorders, with the potential patient population rapidly increasing due to aging [2]. Motor disorders not only significantly impact patients’ daily activities, but impose substantial mental burdens on them [3].

Patients may regain partial or most motor function by rehabilitation training [4]. However, traditional training tends to be tedious, leading to a lack of motivation and engagement, resulting in suboptimal outcomes or abandonment of the rehabilitation process [5, 6]. Motivation and engagement are pivotal for successful rehabilitation [7]. Simple passive repetition is insufficient to enhance motor cortex excitability and lacks benefits for neural remodeling [8–10].

Numerous studies have indicated that integrating virtual reality (VR) games into rehabilitation can enhance patient motivation [8, 11, 12]. Consequently, VR technology has been incorporated into rehabilitation, giving rise to VR rehabilitation. This approach leverages virtual environments tailored to patient needs, fostering interaction and motivation, thereby enhancing rehabilitation outcomes [13]. The past decade has witnessed rapid development in VR rehabilitation, with recent breakthroughs in cost, portability, and reliability of virtual reality technology. This progress positions VR rehabilitation serious games as a burgeoning research focus. Typically, VR rehabilitation serious games utilize commercial motion-sensing games as training content, requiring patients to perform limb movements or balance control to complete tasks [14–16]. While these games encompass physical and cognitive skills, they are originally designed for healthy individuals and may not suit those with motor or cognitive impairments [17]. Consequently, some researchers have designed targeted rehabilitation serious games for specific patient groups [18, 19], integrating game elements into exercises to enhance the enjoyment of rehabilitation training.

Essentially, VR rehabilitation serious games are a type of serious games that leverage VR devices for rehabilitation. Unlike commercial games, serious games do not solely prioritize entertainment; rather, they represent a purposeful integration of games and education. This integration extends to applications in diverse fields such as education, training, rehabilitation, and more, with the aim of enhancing users’ abilities [20].

Despite the promising clinical prospects of VR rehabilitation and the development of numerous VR rehabilitation serious games by scholars, existing research on these games lacks focus on the design and development process [21, 22]. The interdisciplinary nature of VR rehabilitation serious games, involving fields such as rehabilitation medicine, game design, and human–computer interaction, poses challenges to the design process [23, 24]. In the realm of game design, while many researchers have proposed various serious design methods (as detailed in the related work section), serious game design methods specifically tailored for rehabilitation remain insufficient [25]. Furthermore, there is a distinct absence of methodologies within the VR context. In the realm of VR rehabilitation serious game design research, most scholars have predominantly focused on analyzing relevant game elements or limited principles [26–28], with a few proposing design suggestions [29]. There is still a lack of a systematic and specific VR rehabilitation serious game content design method closely integrated with clinical rehabilitation.

In the latest work, Baranyi [25] DeapSea workflow for the design of serious game for stroke rehabilitation. This work outlines the parameters, functions, and workflow essential for implementing rehabilitation serious games from the perspective of a therapist, offering valuable insights for this article. However, the literature lacks a comprehensive introduction to the design process of rehabilitation serious games. Therefore, this paper aims to propose a design framework for VR rehabilitation serious games, offering guidance for their effective design.

The structure of this paper is as follows: the second part provides an overview of existing related work, while the third part introduces the method of how the framework is proposed. The fourth part offers the results of each step introduced in the third part, along with a detailed description of the proposed VR game design framework. The fifth part designs an upper limb VR rehabilitation serious game based on the framework. In the sixth part, another developed VR rehabilitation serious game is analyzed using our framework. Finally, the seventh section concludes with a discussion and summary.

## Related work

### HMD-VR rehabilitation serious games

Due to the breakthroughs in VR technology, numerous scholars have explored the use of head-mounted displays (HMD) for HMF-VR rehabilitation serious game training.

Subramanian [30] contends that feedback aids in the recovery of sensory and motor functions in stroke patients. Accordingly, he developed an HMD-VR rehabilitation serious game featuring exercises and feedback elements. Knowledge of Performance (KP) feedback provides feedback about the motion itself; it focus on process. Knowledge of Results (KR) feedback, on the other hand, refers to feedback that informs patient about the outcome or results of their motion. In this game, patients are required to press virtual elevator buttons on walls with both arms, fostering motor learning through auditory and visual KP feedback, along with KR feedback on motion speed and accuracy. In detail: KP: ‘Whoosh’ sound and red target if trunk displacement exceeds 5 cm. KR: ‘Ping’ sound and green target for successful movements; Negative feedback: buzzer for slow or imprecise movements. However, the developed VR rehabilitation serious game is limited to feedback content and lacks a complete gaming process.

Trombetta [31] designed Motion Rehab AVE 3D, an HMD-VR rehabilitation serious game targeting motivation issues in patients undergoing upper limb and balance training after a mild stroke. The game involves patients standing and stretching their shoulders, elbows, and wrists to catch falling objects and accumulate points. All stretching movements are consistent with traditional rehabilitation requirements, and patients can independently choose difficulty levels through the interactive interface. Despite offering a comprehensive process, the game lacks a design for utilizing earned points for consumption, and there is no integrated storyline.

Weber [32] integrated mirror therapy with HMD-VR rehabilitation, creating a game that generates the illusion of movement exclusively on the affected side. The game consists of two parts: the first involves using the healthy upper limb to stack virtual stones, and the second requires placing plates and glasses in a virtual kitchen. Throughout the process, the game maps the movement of the patient's healthy side to that of the affected limb, creating a mirrored effect. The game action emulates the traditional mirror therapy, offering a total of 30 min of training with an optional rest time at the midpoint. Therapists have the flexibility to select tasks of varying difficulty levels before the game begins. Similarly, by combining mirror therapy with VR rehabilitation, Mekbib [33] developed a bilateral HMD-VR rehabilitation serious game to enhance patient limb coordination. The game entails patients moving their healthy upper limbs to grasp a ball designated by the rehabilitation therapist on the virtual desktop and placing it in a basket. During the process, the healthy side's movement is mapped to simultaneous movements of both upper limbs, aligning the affected side parallel to the healthy side. The game consists of 20 rounds, and after each round, the therapist adjusts the difficulty based on the patient’s performance, progressing to the next game until the 1-h training volume is completed. Both games were designed by the principles of mirror therapy, incorporating game rules, feedback, and the ability to adjust the difficulty, thus transforming mirror therapy into interactive gaming experiences. However, both games lack content to sustain the patient’s long-term training interest.

Despite the development of numerous VR rehabilitation serious games, there remains a deficiency in attention to the design and development process, as well as a lack of in-depth research on VR rehabilitation serious game design methods [17, 21, 22]. Given that VR rehabilitation serious games adopt a gaming approach for rehabilitation, the quality of game design is paramount for the recovery [34, 35]. Thus, there is a need for support from game design approach, which should also be adjusted according to the characteristics of HMD. Consequently, exploring how to design VR rehabilitation serious games represents an important and in-depth area of research.

### Serious game design method

Over the past two decades, numerous scholars have dedicated their efforts to researching serious game design. In this context, our primary focus is on introducing various design methods tailored specifically for serious games.

Hunicke [36] introduced the MDA (Mechanism-Dynamic-Aesthetics) framework, which outlines three fundamental design components of games. These components include (1) Mechanism: this encompasses the fundamental elements of the game at the level of data representation and algorithm, embodying the game’s rules. (2) Dynamics: this pertains to the dynamic behavior of the mechanism in response to player input and system output, evolving over time, and representing the system’s behavior. (3) Aesthetics: this refers to the emotional responses evoked in players through their interaction with the game system, encapsulating the notion of “fun”. In the developmental process of game design, developers typically commence by defining the game’s mechanism, from which they derive corresponding dynamic behaviors of the system. Subsequently, specific aesthetic experiences are crafted based on these dynamic behaviors.

In subsequent studies, numerous researchers have proposed novel game design framework based on the MDA framework. Junior [37] refined the core components—mechanics, dynamics, and aesthetics—within the MDA framework. This endeavor aimed to rectify misunderstand in comprehending MDA, thereby enhancing clarity and facilitating its accessibility for game designers. In contrast, Walk [38] argued that the MDA framework disproportionately emphasizes game mechanics, potentially resulting in a lack of consideration for patient experiences. To address this concern, Walk proposed the Design-Dynamic-Experience (DDE) framework, prioritizing the cultivation of patient-centric experiences. Pendleton [39] proposes a design framework that integrates the MDA framework with Bloom’s Taxonomy. This framework aims to empower individuals with minimal game design backgrounds to achieve greater efficiency in their design. Meanwhile, Winn [40] expanded upon the MDA framework to cater to the requirements of serious game design, proposing the Design-Game-Experience (DPE) framework. Further modifications were made by Cardona-Rivera [41], who adapted the MDA framework for narrative games and introduced the Goal-Feedback-Interpretation (GFI) framework. Additionally, Deterding [42] addressed the need for specific guidance within the MDA framework by proposing a gamification design process. This process revolves around seven Skill Atoms—goal, operation, object, rule, feedback, challenge, and motivation—and employs a series of questions, inspired by Schell's “design lens” [43]. During the process of problem identification, the game design is systematically completed.

Additionally, Kiili [44] introduced an experiential game design model rooted in experiential learning theory and flow theory. However, this model primarily emphasizes providing immediate feedback to players, presenting challenges, and adjusting task difficulty as players progress, thus lacking comprehensive support for the entire game design process. Carvalho [45] proposed a conceptual model for serious game design based on activity theory. This model categorizes serious games into three activities: game activities, learning activities, and teaching activities. Each activity encompasses three game components: actions, tools, and goals, with all game elements situated within these components. The design methodology outlined in this model comprises four steps: describing activities, representing game sequences (game timeline), determining components (actions, tools, and goals), and implementing instructions (identifying which elements are utilized).

De Freitas [46] introduced an educational game design framework primarily structured around four dimensions: usage environment, learner group, game expression, and educational models or methods. Amory [47] extended the original GOM theory and organized it into a framework known as GOM-II, which encompasses multiple game components (objects). Each object possesses both a theoretical (abstract) connection and a specific connection. The theoretical connection links to teaching content and theory, while the specific connection pertains to design elements. Yusoff [48] incorporated nine elements essential for educational games into a comprehensive design framework tailored for educational game development. Silva [49] presented an approach to designing the mechanics of educational serious games. This method delineates all essential steps needed to incorporate learning mechanics into an educational game while distinguishing them from the mechanics responsible for the game’s entertainment value. In recent years, Viudes-Carbonell [50] has introduced an iterative design methodology for the mechanics of educational games, grounded in the Mechanism Dynamic Aesthetics (MDA) framework.

While numerous scholars have proposed various serious game design methodologies, rehabilitation serious games, owing to their patient-centric focus, demand a heightened emphasis on clinical rehabilitation alongside the incorporation of learning skills and entertainment elements. Consequently, although several frameworks or methods exist to guide the design of rehabilitation serious games, many are challenging to directly apply to this specific context. Additionally, despite the numerous studies that have expanded and refined the MDA framework, given its universal applicability, we have chosen to integrate clinic information and VR limitation into the MDA framework. This integration serves as the foundation for our proposed rehabilitation serious game design framework.

### Rehabilitation serious game design methods

In the realm of rehabilitation serious game design, researchers have proposed quite some design principles aimed at enhancing the effectiveness and appeal of games in assisting patients’ recovery.

The study conducted by Burke et al. [26] suggests that to enhance upper limb rehabilitation effectiveness in stroke patients, game design should incorporate meaningful feedback and maintain difficulty levels that align with the patient’s capabilities. Extending this line of inquiry, Doyle et al. [51] delved deeper into the significance of feedback within therapeutic games, specifically examining the impact of visual feedback on patients' accurate execution of exercise actions. Their prototype game demonstrated that real-time feedback could enhance patients’ enjoyment and perception of exercise. However, the study also emphasized the need for further research on diverse feedback types to enhance patients’ confidence and proficiency in performing exercises accurately.

Lohse et al. [52] investigated how video games enhance patient engagement and motivation through their design principles. They argue that key design elements within games, such as choices, rewards, and goal-setting mechanisms, can significantly boost player motivation and engagement, potentially extending the time patients invest in their treatment. Similarly, Shah et al. [53] investigated the impact of various game design parameters, such as operant conditioning and scoring mechanisms, on player intrinsic motivation. Their findings suggested that these parameters can enhance player engagement and enjoyment, highlighting the potential of game design to effectively motivate players. Furthermore, Herne et al. [27, 28] focused on the appeal of virtual reality (VR) for upper limb rehabilitation in stroke survivors. Through user experience case studies, they identified several game design principles that could be crucial for enhancing patient engagement, including awareness, feedback, interactivity, fluency, and challenge. Additionally, factors such as attention, engagement, motivation, effort, clear instructions, usability, interest, psychological absorption, purpose, and first-person perspective were also deemed important.

In the latest work, Baranyi [25] proposed 20 configuration possibilities, 7 functional requirements, and a six-step workflow named DeapSea for the development and design of a novel serious game tailored for stroke rehabilitation. The possible configurations are essentially game parameters that could be modified by therapists. The adjustments of these configurations enable the game to adapt to the individual needs of patients, facilitating personalized treatment. The requirements serve as the essential functions that serious games should offer for therapists’ use. The “DeapSea” workflow encapsulates the entire process of implementing serious game rehabilitation training.

Several studies have approached research from the overarching perspective of rehabilitation serious game design. Lewthwaite [54] advances an OPTIMAL theoretical model from a macro viewpoint, positing that intrinsic motivation and attention can enhance task performance, consequently fostering motor relearning in patients. Similarly, Hashim [55] introduces a dual-task rehabilitation serious game design framework, encompassing game types, game theory, interactive cognitive-motor training techniques, and fundamental game design elements, including challenges, rewards, and feedback. Additionally, Brassel [29] reviews and synthesizes findings from 14 pieces of literature on rehabilitation serious games, offering recommendations for designing rehabilitation serious games across nine dimensions, including rehabilitation principles, adverse reactions and safety considerations, and technological aspects.

Nevertheless, existing frameworks and recommendations still lack systematic and specific guidance concerning rehabilitation serious games. Critical aspects, including the necessary content for designing rehabilitation serious games, considerations for each content aspect, and the essential functional requirements, remain inadequately addressed.

Thus, various studies utilize the Mechanics, Dynamics, Aesthetics (MDA) game design framework in the development of rehabilitation serious games. For instance, Yang [56] utilized the MDA framework to develop a video game aimed at alleviating treatment-related anxiety in children with lymphocytic leukemia. Barathi [57] applied the MDA framework to create a cycling VR rehabilitation serious game. However, challenges arise in the application of MDA to rehabilitation serious game design. Firstly, the abstract nature of the MDA framework can result in misunderstandings and a lack of specific guidance on intricate details, rendering its use challenging [37]. Secondly, MDA’s emphasis on mechanics may not align seamlessly with the priorities of VR rehabilitation serious games, which primarily focus on rehabilitation and emphasize the patient’s movements [38].

Hence, this paper aims to propose a systematic and specific VR rehabilitation serious game design framework that incorporates essential rehabilitation functions and could sustain long-term patient engagement in rehabilitation training.

## Methods

The method is to introduce how the framework is proposed. According to the design theory [58], the process commences with stating the problem (object), followed by information gathering and identifying requirements, and finally generating the game conception.

The aim of VR motor rehabilitation serious games is to assist patients in regaining motion abilities and ensure they remain motivated and engaged during training sessions. Consequently, the objects encompass: (1) Achieving comparable effectiveness to clinical rehabilitation. (2) Enhancing patients’ motivation throughout the training process.

The requirement is the detail of the objects. To identifying requirements, the information should be collected. For the first requirement, how the rehabilitation exercises work should be known, thus, rehabilitation exercises are deconstructed into function blocks. For the second object, to hold patients’ motivation, the game design principles (elements) should be contained. Therefore, relevant literature is analyzed and compared to identify elements that rehabilitation serious games should incorporate to boost patients’ motivation. Furthermore, to further enhance patients’ motivation, VR is introduced.

**Fig. 1:**
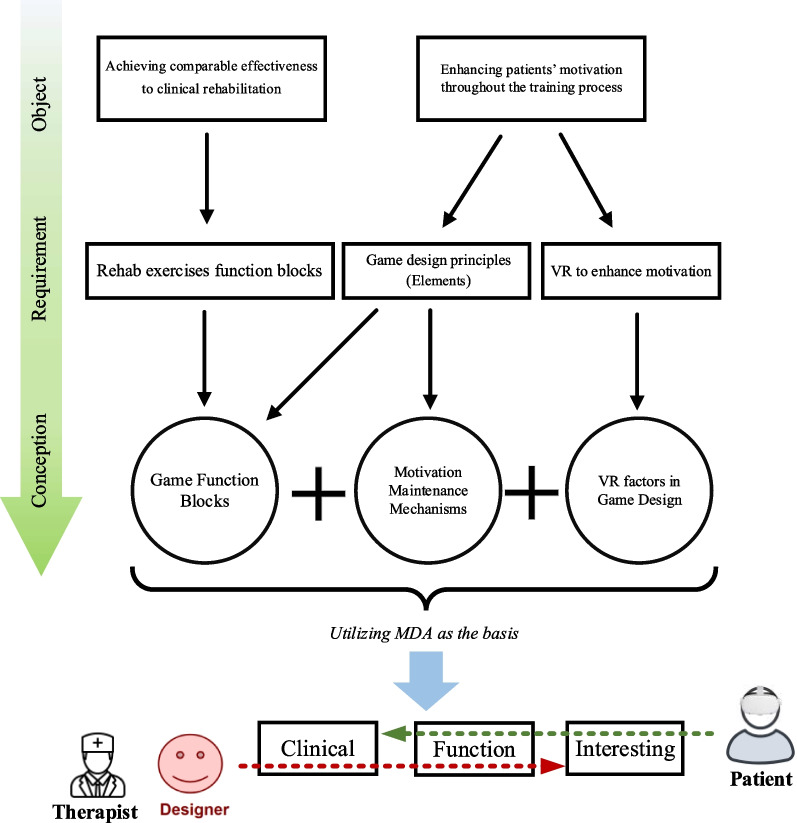
The process of proposing VR rehabilitation serious game design framework

Ultimately, to meet the requirement, the exercise function blocks are initially mapped onto the game design elements, forming game function blocks (for rehabilitation function). Then, based on the game design principles (elements), a reward cycle is proposed to sustain the long-term motivation. Moreover, the VR factors in game design are analyzed. Finally, synthesizing the game elements, VR factors and reward cycle, the framework is proposed. Following the framework, a specific VR rehabilitation serious game can be designed. The workflow of the design process of VR rehabilitation serious game is depicted in Fig. 1.

## Results

In this section, the results of each step in the method, including (1) Analyze and Compare Relevant Literature to identify game elements. (2) Decomposition of clinical rehabilitation exercises and mapping into the game elements. (3) Impact of VR on motor rehabilitation serious games. (4) A reward cycle for long-term motivation, are introduced in detail. The framework is also introduced at the end of this section.

### Analyze and compare relevant literature to identify game elements

The designed game should adhere to the principles of rehabilitation gaming, which must be considered in the proposed rehabilitation serious game design framework. Therefore, relevant literatures are analyzed and compared to identify the elements that games should encompass.

The game elements identified in relevant literatures are listed in Table 1. Furthermore, these elements could be further generalized into following elements: *Feedback, Difficulty, Reward, Goal, Choice, Immersion, Game length, therapeutic principles, Guide* and *Uncertainty*.

**Table 1:** Elements in relevant literature

Related literature	Game elements
Burke et al. [26]	Feedback, challenge
Lohse et al. [52]	Reward Difficulty/challenge Feedback Choice Interactivity Goals Socialization
Herne et al. [27, 28]	Awareness Feedback Interactivity Flow and challenge Motivation and effort Purpose First-person view
Brassel et al. [29]	Maintain therapeutic principles Challenging and customizable Feedback
Barany et al. [25]	Speed, TIME, LIFE Placing/moving objects or obstacles Objects/obstacles behavior and sensitivity Errors Achievements, badges, goals Number of playable levels Difficulty Help Scoring Selections Plausibility
Zain et al. [60]	Challenge Feedback Immersion Adaptivity
Shah et al. [53]	Beating the game Scoring Operant conditioning Feedback
Doyle [51]	Feedback, interaction, customizable

*Feedback* is a common element across all related literature (e.g. *Scoring* in [25]). It includes the KP and KR feedback, which provide patients with information about their motion, enabling them to become aware of their state. Thus, the *Awareness* in [27, 28] could also be categorized under this game element. Additionally, multimodal feedback that response to patient’s motion is also a kind of feedback, presenting the *Interaction* mentioned in [27, 28, 51].

*Difficulty* is also a prominent element across all related literature (e.g. *Beating the game* in [53]). According to the flow theory [59], patients could achieve a state of deep engagement, leading to efficient learning, which requires a balancing personal skill and challenge. Since patients have varying degree of motion function, this balance point differs among individuals. Therefore, *Difficulty* is crucial in rehabilitation serious game to accomplish personalized rehabilitation. As such, *Difficulty, Challenge, Adaptivity* and *Customizable* all refer to the same concept. The method by which *Difficulty* provides suitable difficult to different individuals involves adjusting parameters such as *Speed, Time, Life, Objects/Obstacles behavior and sensitivity*.

*Reward* is referenced in several sources [25, 27, 28, 52], with examples including *Achievements and badges* in [25]. It serves as an incentive structure, giving patient positive feedback in time to encourage continued engagement with training. Therefore, *Motivation and Effort* mentioned in [27, 28] can also be categorized under this game element.

*Goal* is mentioned in various sources [25, 27, 28, 52], such as *Purpose* in [27, 28]. The Goal of the game represents what the designed game intends for patients to strive towards. It could make task (rehabilitation training) execution efficient [52]. Additionally, the achievement of the game goal can offer patients achievement-based satisfaction.

*Choice*, as mentioned in [25, 52]. *Choice* offers patients a sense of control, strengthening their connection to the virtual environment and facilitating active participation in the game.

*Immersion* is discussed in [25, 60], with references to *First-Person View* in [25]. The *immersion* is a kind of feeling, which could be maximally enhanced through VR.

There are additional elements proposed by individual research: *Game length* (*Number of levels*), the total duration time of the game. *Therapeutic principles*, the of rehabilitation approached employed in game. *Plausibility*, the patient could have a rest in time. *Guide* (*Help)*, tell patient what and how to achieve the task. *Uncertainty* (Operant Conditioning), to keep a certain of change in game, which is proved to improve to the motor learning [13].

### Decomposition of clinical rehabilitation exercises and mapping into the game elements

**Fig. 2:**
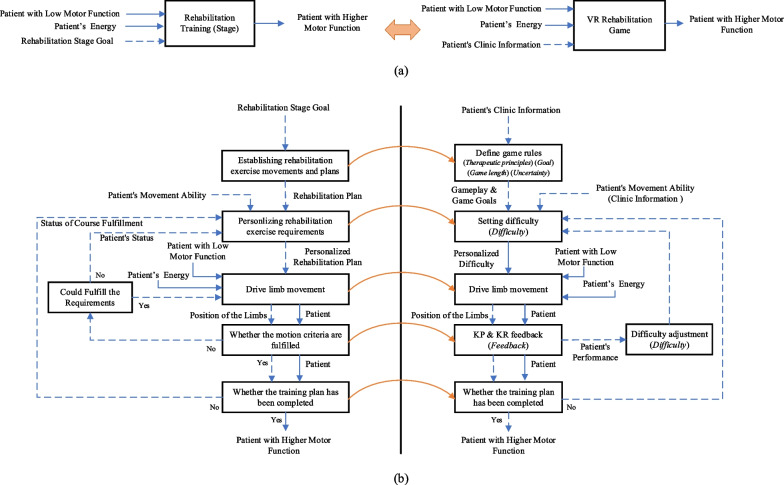
Decomposition diagram of clinical rehabilitation exercises. The solid blue line denotes material and energy, the dashed blue line represents information, and the orange line signifies the mapping

As depicted in Fig. 2a, the clinical rehabilitation exercises (Rehabilitation Training) are represented as a black box (left). Its inputs include Patient with Low Motor Function, Patient’s Energy, and Rehabilitation Stage Goal, while the output is Patient with Higher Motor Function.

The decomposition result of clinical rehabilitation exercises is depicted in Fig. 2b. For clarity, it is presented in a functional decomposition structure diagram. Before commencing the rehabilitation training process, the therapist conducts a clinical evaluation during the initial visit to assess the patient's stage and motor ability. Thus, during the rehabilitation training, the therapist initiates by proposing rehabilitation training actions and plans based on the patient's rehabilitation stage goals. The requirements for rehabilitation training movements are then personally adjusted, accounting for the patient's motor function level. Following this, the patient actively engages in limb movement. If, during the movement, the patient perceives that their motion has not yet met the specified requirements, they persist in driving the limb. In cases where further movement toward the required position proves unattainable, therapists may reduce the training requirements. Upon the completion of a training session, the therapist records the patient's training performance and adjusts the training requirements for subsequent sessions. After the conclusion of the training course, a reassessment is conducted on the patient’s condition, encompassing motor function and ability information.

VR rehabilitation serious game is expected to achieve comparable effectiveness to clinical rehabilitation. Hence, as depicted in Fig. 2a, it should have similar structure to the clinical rehabilitation exercises. Although we cannot achieve a complete functional decomposition result of the VR rehabilitation serious game all at once, it can be partially confirmed by mapping the function blocks of clinical rehabilitation exercises to the game elements identified previously, as illustrated in Fig. 2b.

### VR factors in game design

In addition to the basic functions of clinical rehabilitation training, VR rehabilitation serious games are more important in enhancing patients’ active participation. Therefore, VR rehabilitation serious games utilize head-mounted displays (HMDs) to deliver rehabilitation training. HMDs present scenes in a 3D surround format around the patient, significantly enhancing the aesthetic effect and increasing the patient's immersion. The aesthetic effect primarily involves designing content that patients can directly perceive through their senses in games, including art design and stories, which directly affect the patient’s gaming experience [61]. Therefore, compared with non-VR rehabilitation games, VR rehabilitation games are more likely to provide an enjoyable experience. Moreover, the increased immersion allows patients to better immerse themselves in the game, enabling them to more easily to get emotional changes, such as a sense of purpose, accomplishment, and control, among other emotions. These feelings encourage active participation in rehabilitation serious game.

However, it also introduces side effects [62]. In VR rehabilitation, patients may encounter visual fatigue and motion sickness. HMDs leverage the disparity between the left and right eyes to present a three-dimensional image. The magnitude of this disparity influences the depth of the image, and a larger disparity can lead to a more pronounced inconsistency in the patient's binocular focus and adjustment, contributing to visual fatigue. Additionally, the HMD’s display is in close proximity to the patient’s eyes, and prolonged focus on the screen can necessitate constant adjustments, further intensifying visual fatigue [63]. Therefore, it is crucial to control the duration of single-round games, allowing patients’ eyes to rest periodically.

The primary cause of motion sickness in VR rehabilitation serious games is sensory conflict. As all mapped motions come from the patient’s movements instead of a controller in VR rehabilitation, the primary factor contributing to motion sickness is latency caused by the device. Studies indicate that delays of less than 15 ms can effectively mitigate the occurrence of motion sickness [64]. Hence, in HMD-VR rehabilitation serious games, it is essential to prioritize the use of preset models (such as cylinders and cubes) to minimize complex models. This approach could alleviate the CPU computation and GPU rendering burden, consequently reducing latency, and addressing motion sickness concerns.

### A reward cycle for long-term motivation

However, as rehabilitation training spans a considerable duration, the enjoyable aspects provided by HMDs alone may not be sufficient to sustain enduring interest among patients. Consequently, a long-term motivation promotion mechanism is necessary.

A reward mechanism is popular among rehabilitation serious games. It emphasizes the significance of the reward, as it could encourage patients to engage to get the reward in short-term. However, in a long-term, as patients receive numerous rewards, the allure of these rewards may diminish, leading to a decline in motivation.

**Fig. 3:**
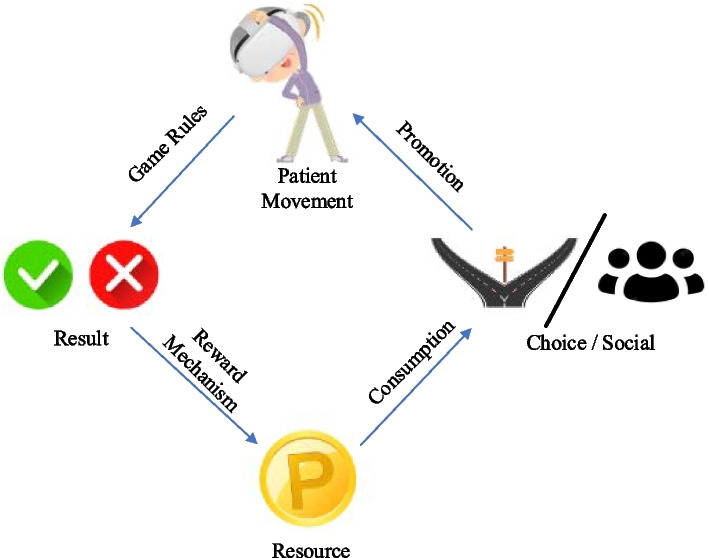
Reward cycle designed to sustain the patient’s long-term training cycle. Following the patient's exercise, game results (success or not) are determined based on the game rules. The reward system then offers resource rewards for the patient’s success. Upon accumulating a certain level of resources, these can be consumed, empowering patients to make independent choices or engage in social with others, thereby further encouraging the patient’s exercise

Therefore, we propose a reward cycle, emphasizing the consumption part to promote the long-term motivation. As illustrated in Fig. 3, after the patient’s exercise performance, game results (success or failure) are determined based on established rules. The reward system gives patients resources, such as points and coins, for the patient’s successful game results. Upon accumulating a certain level of resources, patients can consume these resources to make selections or engage socially with others. This includes spending points on new interaction goals, entering rankings, and competing with others. This establishes a cycle of “*patient movement* $\stackrel{Game rules}{\to }$ *Result* $\stackrel{Reward System}{\to }$ *Gain Sources *$\stackrel{consumption}{\to }$* Choice/Social *$\stackrel{Promotion}{\to }$* patient movement*”. In this cycle, if the patient fails to reach the next step—such as failing to achieve success in exercise or having insufficient resources for consumption—it amplifies the patient’s desire to progress to the subsequent stage. This represents external motivation, motivating them to exert greater effort. Once a cycle is completed, the choices and social interactions meet the patient's individual or relational needs, enhancing the patient's intrinsic motivation and fostering a commitment to long-term rehabilitation training.

Indeed, the proposed loop builds upon a single reward by introducing another that necessitates prolonged effort from the patient. Thus, akin to a single reward, the attractiveness of the proposed cycle may diminish with many times repeated iterations. Hence, the cycle period should not be excessively short. However, if the time required to achieve the “long-term reward” is excessively long, it may seem unattainable for patients, thus failing to produce the desired effect. Hence, it’s crucial to appropriately control the length of the cycle period, with the optimal period possibly varying depending on individual personality traits. Additionally, as rehabilitation aspects required time is varies, the design of this cycle should ensure that (Number of cycles) * (Cycle period) ≥ (Rehabilitation duration).

### VR rehabilitation serious game design framework

**Fig. 4:**
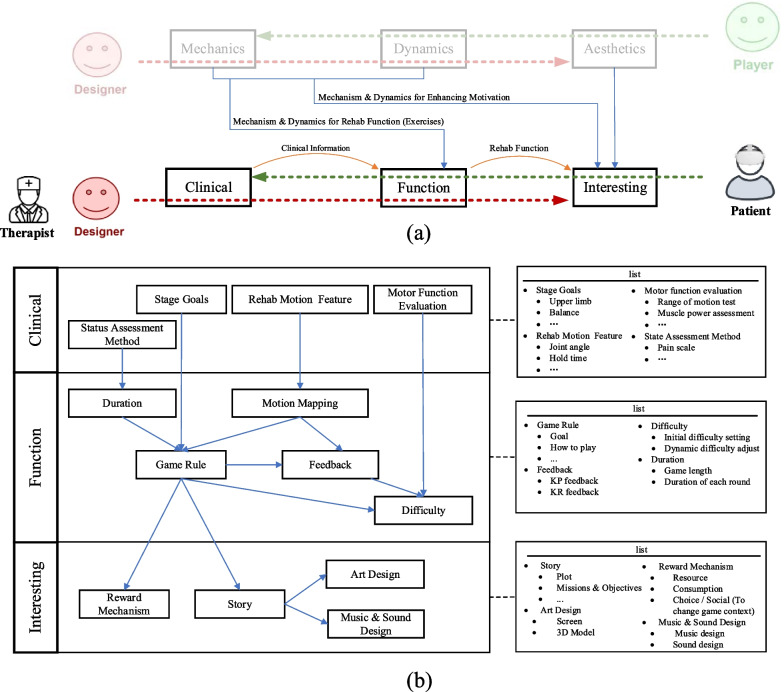
VR Rehabilitation serious game Design Framework. **a** The overall structure of the VR rehabilitation serious game design framework. **b** Details the specific process of designing VR rehabilitation serious games. Clinical information, serving as a clinical representation, is positioned closer to the designer and rehabilitation therapist. “Interesting” represents the content directly experienced by players and is closest to them. “Function” signifies the mapping of the functions of rehabilitation to game function, acting as a bridge between the “clinical” and “interesting” aspects

Combining the game function blocks (for rehabilitation function), VR factors, and reward cycle for long-term motivation, this paper presents a comprehensive design framework: Clinical Function Fun (CFI), as depicted in Fig. 4.

Giving priority to clinical rehabilitation is essential in VR rehabilitation serious games. This process involves initially considering clinical rehabilitation motions, then determining the mapping of patient movements in the game based on these clinical movements, and ultimately structuring the game around patient movements. The MDA framework, where mechanics take precedence in game design, proves inadequate for meeting the specific design requirements of VR rehabilitation serious games. In response to this challenge, this paper introduces a novel VR rehabilitation serious game design framework: Clinical Function Fun (CFI). “Clinical” is the closest to the designers and therapists, serving to determine clinical information to guide game design. Subsequently, guided by this clinical information, the game “Function” is designed, to align with the fundamental functions of rehabilitation. Ultimately, “Interesting” is designed to sustain patients’ engagement in long-term rehabilitation training.

#### Clinical

VR rehabilitation serious games must be designed with a clinical perspective to ensure alignment with clinical requirements. The term “clinical” here pertains to providing clear information relevant to clinical practice, encompassing the patient’s stage goals, features of clinical rehabilitation exercises, evaluation of the patient's motor function and the assessment of patient’s state. Within this, the patient’s stage goal primarily aims to clarify the rehabilitation content needed by the patient at the current stage, providing direction for game rule design in “function.” For instance, in patients with mild to moderate stroke (patient stage), the goal may be to train the overall coordination function of the upper limbs (objective). The features of clinical rehabilitation motions involve breaking down the patient's rehabilitation motions into specific indicators, such as joint angle and posture retention time, which are instrumental for the later design of motion mapping in “function.” The assessment of patient motor function is to address the requirements of “difficulty” within the “function” aspect to accomplish personalized rehabilitation for patients. The state assessment method is employed to identify the patient’s current condition, such as using a pain scale, to determine the duration of the rehabilitation training within the game. Unlike motor function assessment, which is conducted only before and after the game, state assessment needs to be performed multiple times during the game process to determine when to conclude the game session.

#### Function

“Function” signifies the functionality of VR rehabilitation serious games, constituting the mapping of fundamental rehabilitation functions within the game. While “Clinical” encompasses information derived from therapists’ diagnoses and “Interest” represents the patient’s direct experience, the purpose of “Function” is to establish a relationship between them. Within “Function,” various components are incorporated, including the design of motion mapping, duration design, game rules, feedback mechanisms, and methods for setting and adjusting difficulty.

Movement mapping translates the rehabilitation motions that patients need to perform in a clinical setting into the content of VR rehabilitation serious games. More specifically, it is based on previously identified features of clinical rehabilitation motions, determining which patient movements will affect the virtual gaming world. In VR rehabilitation serious games, motion mapping plays a foundational role in the functional layer, influencing the design of game rules, and serving as the basis for KP feedback. Indeed, in the movement mapping process, ensuring adequate space for patients’ movements is paramount. In a virtual environment where patients cannot perceive the real world, there’s a risk of collisions or other hazards if the context is not carefully monitored and controlled.

The duration serves for goal in game rules. It can be determined by evaluating the patient’s current state or by setting predefined criteria, aiming to fulfill the required training volume as practiced clinically, such as “as often as possible,” “x times,” or “only if no pain is experienced.” Additionally, considering the potential side effects of VR devices, such as visual fatigue, it is essential to set the duration of single round games to allow patients to take timely breaks when needed.

The game rules constitute the fundamental content of game operation, primarily focusing on establishing the goal and gameplay. The goal is to gamify the duration of the game, specifically by translating it into in-game parameters such as the number of targets or the remaining time. The gameplay entails gamifying the previously mapped motion content while considering the stage goals of clinical, thereby determining relevant game parameters concurrently. Additionally, uncertainty, which means a certain of change should be considered in gameplay.

The feedback system in the game serves as the response to a patient’s actions, providing information about the impact of their actions on the virtual world. Feedback comprises performance-based (KP) feedback and result-based (KR) feedback. Knowledge of Performance (KP) feedback provides feedback about the motion itself; it focus on process; therefore, it needs to be designed based on motion mapping. Knowledge of Results (KR) feedback, on the other hand, refers to feedback that informs patient about the outcome or results of their motion, necessitating design considerations based on the game objectives outlined in the game rules. Feedback can be achieved through multiple modalities including visual, auditory, and tactile senses.

According to the flow theory [59], individuals can achieve a state of deep engagement and forgetfulness, leading to efficient learning, which requires a balancing personal skill and challenge. This theory led to the development of Dynamic Difficulty Adjustment technology (DDA), which is incorporated as a component of Difficulty in the proposed framework. The design of difficulty encompasses both the initial difficulty setting and adjustment, playing a crucial role in personalized rehabilitation for patients with varying degrees of injury. This element ensures that the difficulty levels always align with patients’ motor abilities, preventing them from feeling discouraged or bored. The difficulty setting and adjustment methods primarily revolve around the relevant parameters outlined in the game rules, making the design of difficulty inherently dependent on the design of these rules. The initial difficulty setting is established based on the clinical evaluation results of the patient’s physical ability. On the other hand, dynamic difficulty adjustment is rooted in the patient’s performance during the training process. This dynamic adjustment involves modifying the parameters defined in the game rules to ensure that the difficulty level aligns with the patient's physical and mental state.

#### Interesting

Like the aesthetics in MDA, the initial impression patients have in VR rehabilitation serious games is centered around whether the game is interesting. Consequently, “Interesting” becomes the element closest to the patient. This part primarily encompasses reward mechanisms, game stories, art design and music & sound design.

The reward mechanism is an incentive structure to sustain long-term training for patients, encompassing resources, consumption, and choices/socializing. Resources, often represented as points or coins, are acquired by patients upon achieving their goals, dependent on the design of game rules. Consumption involves utilizing acquired resources to modify game content, with choices or social interactions serving as specific means. In the context of choices, patients can independently make decisions that impact the game content, fulfilling their need for self-expression. Social interaction allows patients to engage with others in the game, fostering collaboration or competition and meeting their relational needs. By addressing the patient's self or relationship needs, intrinsic motivation is enhanced, facilitating long-term engagement in gaming training.

The game story is an extension of the game rules, presenting them to patients in the narrative. This allows patients to perceive the meaning and mission of the motions, immersing themselves in the experience. The game story incorporates various elements, with missions and objectives derived from the goals and gameplay outlined in the game rules. Missions, composed of multiple objectives, are further wrapped by the story to enhance the patient’s immersion. Moreover, a clear guide should be contained in the story.

The art design is grounded game story, shaping scenes and objects to provide patients with direct sensory stimulation. Moreover, the chosen art style plays a crucial role in determining the game atmosphere. Therefore, it is imperative to consider a style suitable for the target patient group during the design process. Additionally, when utilizing HMD, especially wireless HMD, it is advisable to employ preset models to minimize CPU computation and GPU rendering burden, thereby reducing latency.

Music and sound design involve creating background music (BGM) and sound feedback in Function-feedback. In rehabilitation serious games, pleasant sound effects are often utilized to reinforce positive feedback, thereby boosting patient motivation. Meanwhile, music design aims to enrich the game atmosphere to enhance patient immersion in the gaming experience. Therefore, the designed music should align with the game’s narrative to ensure consistency and enhance the overall gaming experience.

## Game design: a case

**Fig. 5:**
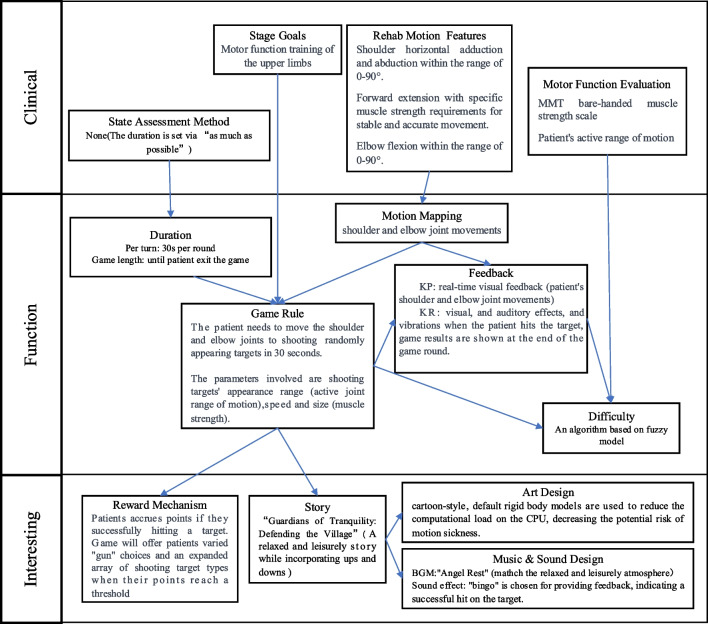
The utilization of the proposed framework in the game design process

Utilizing the proposed VR rehabilitation serious game design framework CFI, this paper develops an upper limb HMD-VR rehabilitation serious game tailored for patients with mild to moderate stroke as an example. The game is developed via the Unity3D game engine. The utilization of the proposed framework in the game design process is illustrated in Fig. 5.

### Clinical

The “Clinical” part is to get clear information relevant to clinical practice, encompassing the patient’s stage goals, features of clinical rehabilitation exercises, evaluation of the patient’s motor function and the assessment of patient’s state.

The upper limb VR rehabilitation serious game is developed for patients with mild to moderate stroke (Brannstrom levels IV and V). This category includes patients capable of some degree of upper limb gross functional autonomous movement but unable to complete most autonomous movement tasks. Additionally, it encompasses patients who are approaching a healthy state but still exhibit insufficient upper limb coordination. The targeted patients require comprehensive upper limb motor rehabilitation, focusing on the shoulder and elbow joints. Consequently, the stage goal is to facilitate the overall motor function training of the upper limbs.

Considering the clinical rehabilitation motions of mild to moderate stroke patients as outlined in Table 2, the shared features of rehabilitation motions for Brannstrom IV and V grade patients include:

- Shoulder horizontal adduction and abduction within the range of 0–90°.
- Forward extension with specific muscle strength requirements for stable and accurate movement.
- Elbow flexion within the range of 0–90°.

**Table 2:** Clinical rehabilitation motors and its features of Brannstrom IV and V patients [65]

Class	Motion	Feature	Illustration
Brannstrom IV	Raising upper limbs	Shoulder extension from 0° to 90°	
	Elbow flexion and extension	Elbow flexion from 90° to 0	
	Suspended hold	Muscle strength (movement stability and accuracy)	
	Shoulder flexion	Shoulder flexion from 0° to 90°	
	Shoulder abduction	Shoulder abduction from 0° to 90°	
	Height-reaching training	Shoulder flexion as high as possible	
Brannstrom V	Shoulder abduction with elbow rotation	Shoulder abduction from 0° to 90°; Elbow rotation from 90° to 90°	
	Wrist extension	Wrist joint extension	
	Grasping and releasing small ball	Coordinated movement	

The evaluation results of motor function are obtained during the first visit diagnosis and will be used for subsequent initial difficulty setting. Based on the features of related clinical rehabilitation motions, in this game, the primary considerations include the patient's muscle strength scale (MMT bare-handed muscle strength scale) as well as the patient’s active range of motion in the shoulder and elbow joints.

### Function

“Function” represents the functionality of VR rehabilitation serious games, constituting the mapping of fundamental rehabilitation functions within the game. According to the proposed farmework, the components in “Function” are designed based on “Clinical”.

The design of motion mapping is designed based on the rehabilitation motion features. Consider the rehabilitation motion features in clinical practice: shoulder adduction and abduction 0–90°, elbow flexion 0–90°, and stable and accurate movement. Hence, the patient's shoulder and elbow joint movements will be mapped into the game.

Based on this motion mapping and stage goal of overall upper limb motor function training, a gameplay in which the patients drive their shoulder and elbow joints to shoot targets is designed. Considering the desire for patients to exercise as much as possible, the game length is designed to provide patients with the option to exit the game whenever they feel fatigued. Considering the possibility of visual fatigue caused by HMD, a game goal of moving the upper limbs as much as possible within 30 s per game round is designed. Therefore, the designed shooting game rules: the shooting target will randomly appear in front, and the patient needs to move the shoulder and elbow joints to accurately align the gun (upper limb end) with the target. The parameters involved are shooting targets’ appearance range (active joint range of motion) and speed & size (muscle strength). Each round of the game lasts for 30 s.

**Fig. 6:**
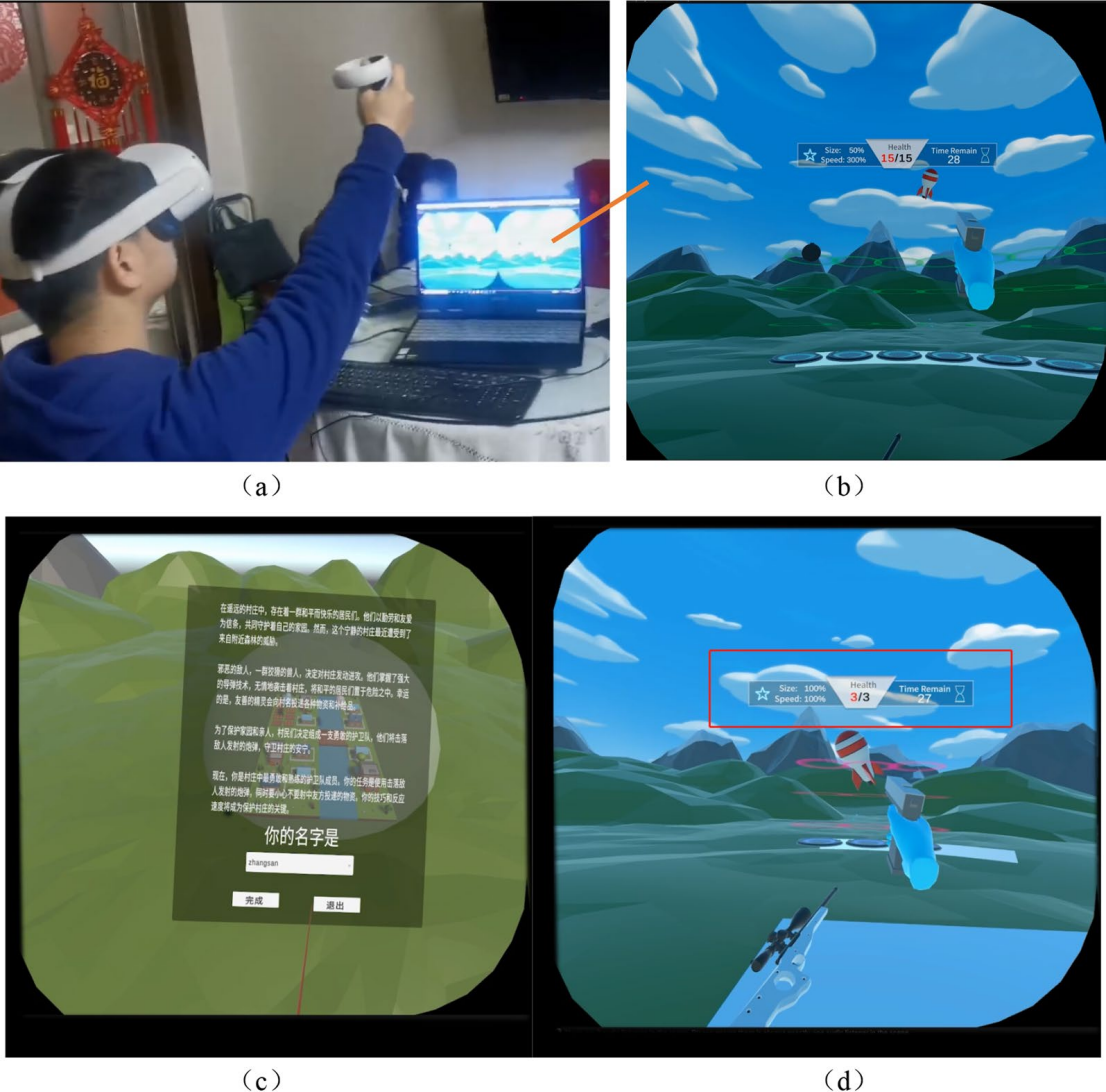
Screenshot in the game. **a**, **b** The mapping of real-world actions in the virtual environment and the corresponding feedback from the virtual world. **c** Story description and task introduction within the game. **d** patient’s perspective in game. The red box presenting crucial game information. This includes, from left to right, details such as the size and speed of missiles/supplies, the current health level/upper limit of the village, and the remaining time in the current round of the game

The patient’s shoulder and elbow joint movements will be feedback to the patient in real-time through visual feedback based on the position information of the hand model (KP feedback), as shown in Fig. 6a, b. At the same time, when the patient accurately hits the target, they will receive feedback on visual, and auditory effects, and tactile vibrations (KR feedback). In addition, game result (one round) will be presented to patients in the form of success rate, helping patients intuitively know their performance (KR feedback).

**Fig. 7:**
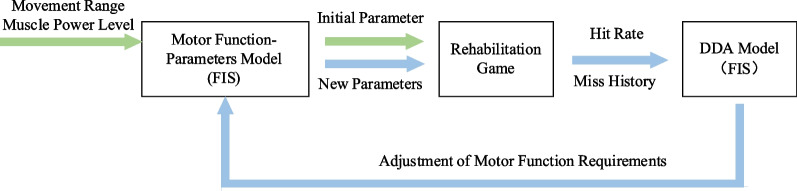
VR the flowchart for difficulty setting and adjustment. The procedural sequence initiates with a clinical evaluation, yielding motor function requirements. Initial parameters are established through the ‘Motor Function-Parameters Model.’ After a gameplay session, performance data is recorded and input into the dynamic difficulty adjustment algorithm. This algorithm computes the adjustment amplitude for the generated motion function requirements, allowing for the modification of motor function requirements. The refined motion function requirements are subsequently reintegrated into the ‘Motor Function-Parameters Model,’ thereby generating updated difficulty parameters

The initial setting and dynamic adjustment of difficulty are the basis for improving patient training participation and motivation. Therefore, this paper proposes a difficulty setting and adjustment algorithm based on the parameters designed in the game rules and the clinical evaluation of patient motor ability. The process is shown in Fig. 7, which includes a game difficulty setting model and a dynamic difficulty adjustment algorithm based on patient performance.

**Fig. 8:**
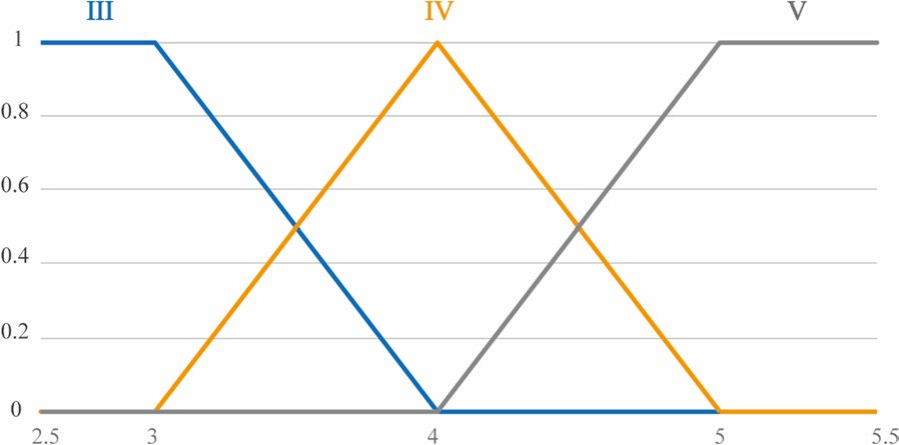
Fuzzy sets and their divide. Blue, yellow, and gray denote three fuzzy sets: III, IV, and V. The horizontal axis signifies muscle strength requirements, while the vertical axis represents the probability of belonging to a specific fuzzy set. Post MMT clinical evaluation, the recorded results yielded grades 3, 3, and 4, respectively. Consequently, on the horizontal axis (muscle strength requirement), the figure reflects a value of 3.33. Correspondingly, on the vertical axis, the probabilities for fuzzy sets are as follows: Fuzzy Set III at 0.67 and Fuzzy Set IV at 0.33. Utilizing these probabilities, the game parameters (the size of the shooting target), corresponding to each fuzzy set, are weighted, and aggregated to derive the design target size

Initially, the evaluation results of motor function in the “Clinic” contribute to acquiring the patient’s joint motion range and muscle strength level. Moreover, the appearance range of the shooting target can be computed based on the determined joint motion range. Nevertheless, the qualitative nature of the Manual Muscle Testing (MMT) scale poses challenges in directly aligning with game parameters. Therefore, a fuzzy method is utilized here. In detail, the designed game incorporates fuzzy sets and membership functions, establishing a nuanced relationship between MMT scale outcomes and target size, illustrated in Fig. 8. Consequently, the initial difficulty level of the game is generated, utilizing insights derived from the clinical evaluation results of motor function.

**Fig. 9:**
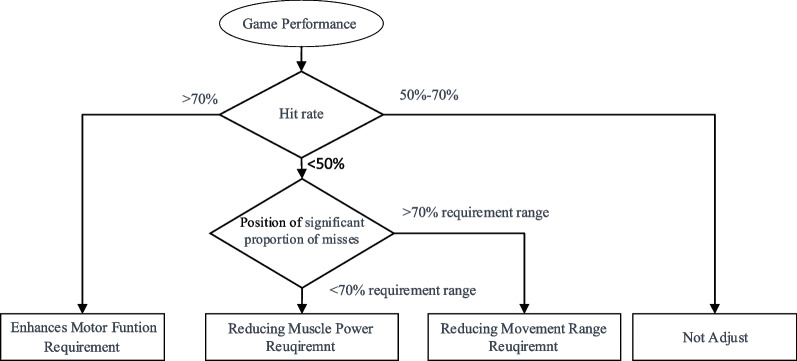
The flowchart for Dynamic Difficulty Adjustment algorithm. The decision to adjust difficulty is determined based on the hit rate. When a difficulty reduction is deemed necessary, a thorough analysis of the spatial distribution of missed targets becomes essential. If a significant proportion of misses is observed beyond the required range by 70%, it is deduced that the primary factor contributing to the low hit rate is the high requirement for motion range. Thus, reducing the movement range requirement. Conversely, if most misses occur within the acceptable range of motion, the predominant factor is likely muscle strength requirements. In such cases, the muscle strength requirement is reduced

After setting the initial difficulty level of the game, a dynamic adjustment becomes essential, tailoring the difficulty according to the patient's performance. Consequently, the establishment of a dynamic difficulty adjustment system is imperative, ensuring continuous adaptation based on the evolving capabilities of the patient. Given that rehabilitation serious games address two critical indicators—patient range of motion and muscle strength—it becomes necessary to discern which indicator requires adjustment. Moreover, the uncertain aspect of determining the quantity of parameter values requiring adjustment adds complexity to the system. To address these challenges, the designed game incorporates the fuzzy module once again to craft a dynamic difficulty adjustment algorithm, intricately responsive to patient performance, as illustrated in Fig. 9.

This paper draws upon the psychological model established by Cameirao [66], grounded in the Yerkes-Dodson law. According to this model, when the patient's hit rate falls below 50%, the Dynamic Difficulty Adjustment (DDA) system intervenes by adjusting the game difficulty to an easier level. Conversely, if the hit rate surpasses 70%, the DDA system enhances the game difficulty to present a higher challenge.

For determining the parameter requiring adjustment, the DDA system notes instances where the patient fails to hit the target before it disappears. If a significant proportion of misses are observed beyond the required range by 70%, it is deduced that the principal factor contributing to the low hit rate is the high requirement for motion range. As a result, the system takes corrective action by reducing the specified motion range requirement. Conversely, if the misses are linked to inadequate muscle strength, the DDA system implements adjustments by lowering the muscle strength requirements.

**Fig. 10:**
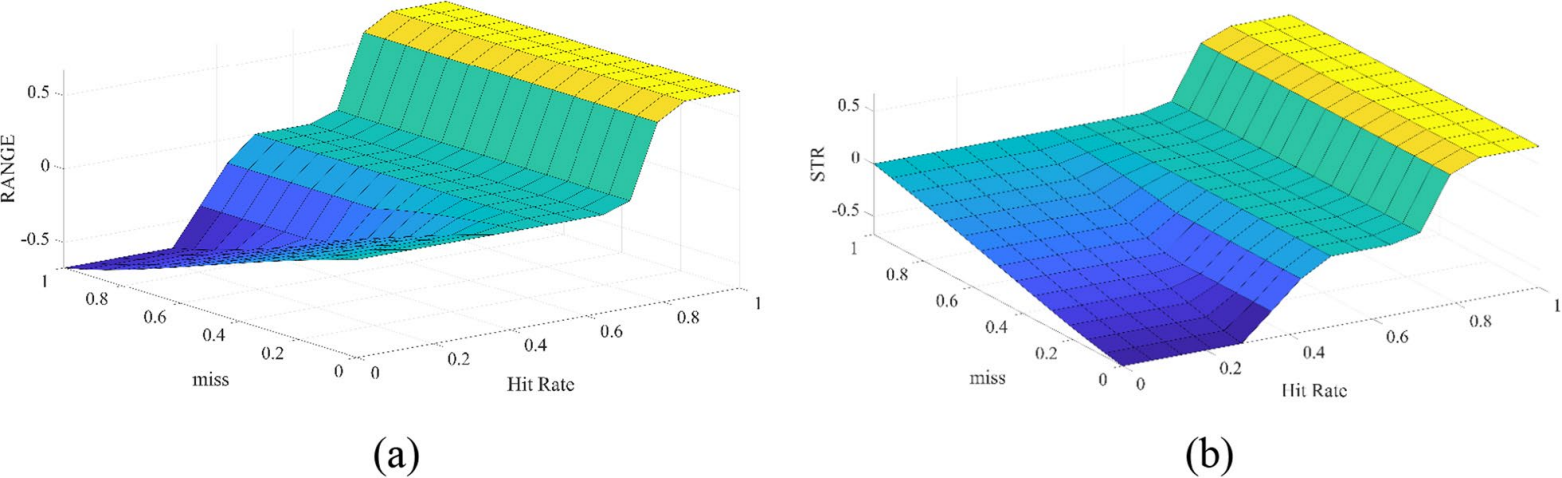
The surface of DDA-FIS. **a** The surface map of dynamic adjustment in Movement Range. The x-axis coordinate denoted as “hit rate,” ranging from 0 to 1. This signifies the percentage of successfully hit targets about all targets. The y-axis coordinate, labeled "miss," also ranges from 0 to 1, representing $$\frac{miss beyond 70\mathrm{\% }requirement}{The number of total miss}$$, If the miss targets are all outside the 70% range, i.e. 1, it indicates that the range of motion requirement factor is the main cause of miss. Conversely, it indicates that muscle strength requirement is the main cause of miss. The Z-axis coordinate represents the adjustment amplitude required for the range of motion, representing the percentage of maximum step size adjustment. **b** The surface map of dynamic adjustment in Muscle Power. The x-axis and y-axis is similar to the surface map of dynamic adjustment in Movement Range. The Z-axis coordinate represents the required adjustment amplitude for muscle strength and also signifies the percentage of maximum step size adjustment

A fuzzy module is employed to construct a rule library based on the previously defined rules to determine the adjustment values. Specifically, the fuzzy module operates as a dual-input, dual-output module, with variations in two motion function requirements. The inputs to this module include the proportion of missed targets outside the required range by 70% and the overall hit rate, while the outputs represent changes in the patient's motion function requirements. The outcomes of the fuzzy module are illustrated in Fig. 10. It is evident that when the hit rate falls within 50%–70%, the parameters remain unchanged. Conversely, when the hit rate surpasses 70%, the system mandates an increase in requirement. In contrast, if the hit rate is below 50%, a reduction in the motion function requirement takes place, contingent upon the position of the missed target.

Input the determined amplitude of changes in patient motor function requirements into the initially established “clinical indicator-game parameter” relationship model to derive specific adjustment values for game parameters.

### Interesting

“Interesting” is the part closest to the patient. True to its name, it aims to enhance the game's appeal and engage patients effectively, building upon the “Function”. This part primarily encompasses reward mechanisms, game stories, art design and music & sound design.

Building upon the established game rules for the preceding “functions,” a reward mechanism has been implemented to sustain long-term rehabilitation training for patients. Upon successfully hitting a target, patients accrue points. Upon accumulating a designated point threshold, the game will offer patients varied “gun” choices and an expanded array of shooting target types. Distinct “guns” are endowed with unique special effects and impacts. Nevertheless, these effects come with corresponding adjustments in point weight. For example, selecting a sniper rifle results in a deceleration of game speed (excluding movement mapping and bullet speed), concomitantly reducing the points earned from hitting targets. Likewise, diverse shooting target types display varying speeds and points.

Thus, a cyclic process is established: “patient engages in moving shoulder and elbow joints to aim at the shooting target $\stackrel{\mathrm{game rules}}{\to }$ feedback $\stackrel{\mathrm{Reward Mechnism}}{\to }$ Gain Points $\stackrel{{\text{Consumption}}}{\to }$ unlocking diverse “gun” choices and shooting target models $\stackrel{{\text{promotion}}}{\to }$ patient engages in moving shoulder and elbow joints to aim at the shooting target”.

Considering that stroke patients, primarily aged 50 and above [67] may encounter difficulty in accepting overly intense storylines and exaggerated effects. Hence, the designed game story is structured to establish a relaxed and leisurely atmosphere while incorporating ups and downs to evoke the patient’s emotional responses and motivate active participation in the training process.

Indeed, with the advancement of Large Language Models (LLMs), various text-related tasks, including storytelling, can be effectively addressed. CHATGPT is such a kind of LLM model created by OpenAI. It can harness the power of machine learning and natural language processing to generate coherent and logical text based on the given prompts or context. Thus, building upon the game rules outlined in the “Features” section, input “Write a relaxed and leisurely shooting game plot with some ups and downs” to CHATGPT and leverage its results to design a storyline centered around “shooting down bombs to defend the village.”

Incorporating the designed storyline, the game scene is envisioned to be set amidst picturesque mountains and quaint villages. The shooting target is represented as shells threatening the villages, as illustrated in Fig. 6. The shells are artistically crafted in various cartoon styles, adding a delightful element to the gameplay. As players accumulate certain points, they unlock access to various cartoon-style shells, further enriching the patient experience. The shell models are designed with shapes closely resembling cylinders or spheres. This design choice eliminates the necessity for intricate triangular mesh calculations for the rigid body, enabling the direct use of a default rigid body model. This optimizes performance and reduces the computational load on the CPU, decreasing the potential risk of motion sickness.

To match the relaxed and leisurely atmosphere of the village storyline, the serene music track “Angel Rest” from the game “NEEDY GIRL Overdose” [68] was selected as the BGM. In “NEEDY GIRL Overdose”, the playback speed of the BGM can be altered to reflect the level of pressure on the game character. Similarly, in our game, variations in the music playback speed are utilized to underscore the story’s peaks and valleys. Specifically, when a patient misses a target (i.e., the target ascends to its highest point and disappears), the BGM playback speed is increased by 10%. Additionally, the "bingo" sound effect is chosen for providing feedback, indicating a successful hit on the target.

## Applied in other VR rehabilitation serious game

**Fig. 11:**
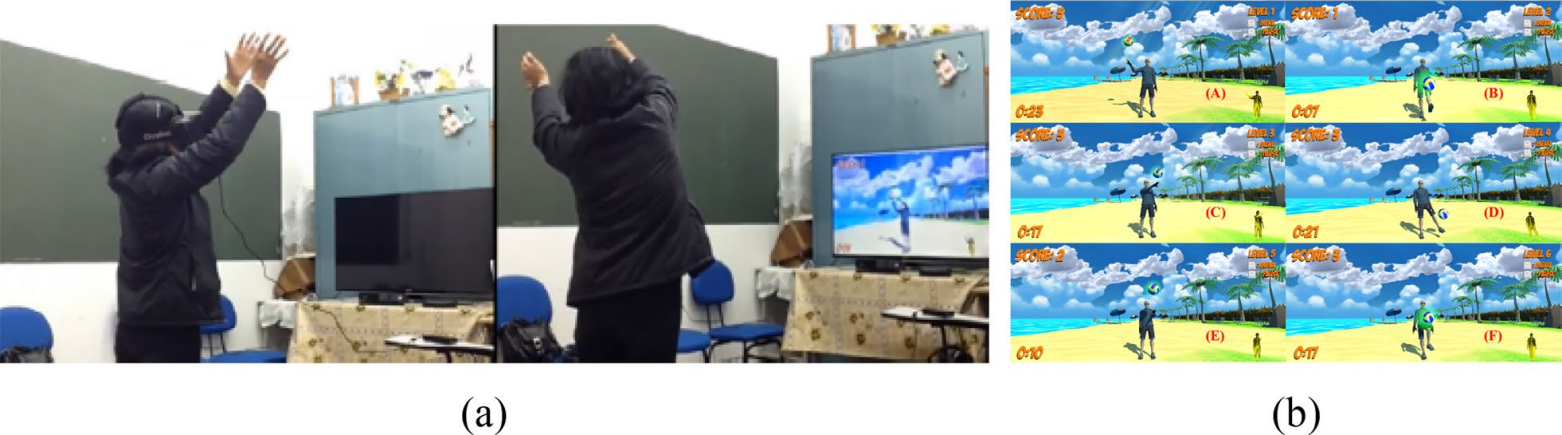
**a** Participants during the task with HMD (left) and Smart TV 3D (right). **b** Screenshots of the Motion Rehab AVE 3D exercises (adopted from [31])

To further elucidate the framework, it is aligned with other VR serious game. Motion Rehab AVE 3D [31] is a VR rehabilitation serious game designed for the post-stroke rehabilitation of patients with mild stroke, as shown in Fig. 11. The primary goal is to provide a novel technology that complements traditional therapy and motivates patients to engage in their rehabilitation program under the supervision of healthcare professionals.

**Fig. 12:**
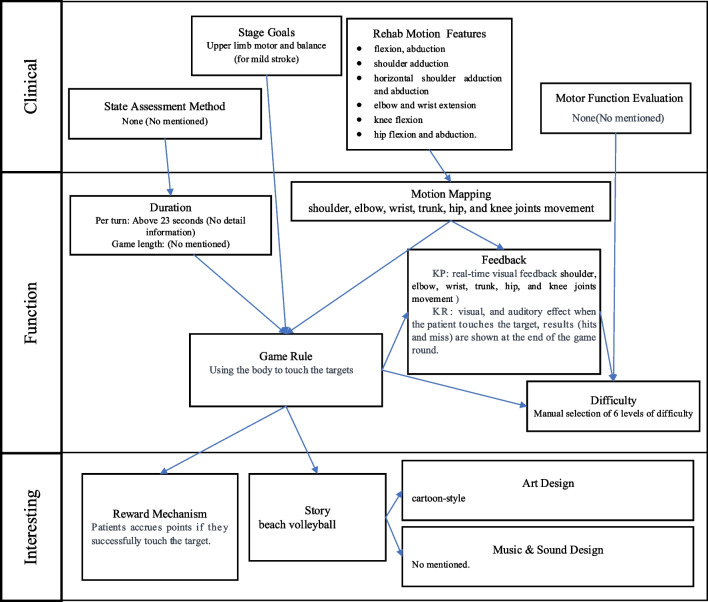
The application of the proposed framework aligned with the Motion Rehab AVE 3D

The application of the proposed framework aligned with the Motion Rehab AVE 3D is illustrated in Fig. 12. Based on the proposed framework, the initial step is “Clinical” part, which aims to gather clinical information necessary for game design. Motion Rehab AVE 3D is specifically designed to aid patients with mild stroke in upper limb and balance rehabilitation. It focuses on six exercises: flexion and abduction, shoulder adduction, horizontal shoulder adduction and abduction, elbow and wrist extension, knee flexion, and hip flexion and abduction. This elucidates the elements “Stage Goals” and “Rehab Motion Features”. However, the article lacks further information about the “State Assessment Method” and “Motor Function Evaluation”. It only briefly mentions the consideration of patient fatigue, leading to a two-minute break between activities.

Based on the information in “Clinical”, the elements in the “Function” part can be further designed. The “Rehab Motion Features” reveal that in “Motion Mapping,” shoulder, elbow, twist, trunk, hip, and knee joint movements need to be mapped. However, the article overlooks the consideration of context in real world when utilizing VR. Additionally, the “Duration” element, referring to game length, is not explicitly addressed in the article. Based on “Duration,” “Stage Goals,” and “Motion Mapping,” the “Game Rules” are devised to involve touching the target with the body within a limited time. Accordingly, “Feedback” is established. Motion Rehab AVE 3D incorporates real-time visual feedback (KP) for shoulder, elbow, twist, trunk, hip, and knee joints, along with visual and auditory feedback upon successful target contact, and feedback on the number of hits and misses after the round of the game (KR). Regarding “Difficulty”, Motion Rehab AVE 3D offers six preset difficulty levels for patients to choose from, rather than adjusting based on individual performance and motor function, which may lead to mismatch with patient’s motor function.”

Finally, the “interesting” part could be designed following the “Function” part to enhance patients’ experience. In the “interesting” part, “Reward Mechanism” element can be established based on the “Game Rules.” Motion Rehab AVE 3D incorporates a scoring reward mechanism, allowing patients to earn points upon successfully touching the target. However, this reward mechanism lacks consumption component. Consequently, in cases of long-term rehabilitation training, once the score no longer provides a sense of achievement, patient’s motivation may diminish. Moreover, leveraging the “Game Rules”, the “Story” element can be further developed to augment immersion. However, the article does not detail the storyline, only hinting at it being beach volleyball through Fig. 11. Based on “Story”, “Art Design” and “Music & Sound Design” elements can be designed. As depicted in Fig. 11, the “Art Design” adopts a cartoon style, while the article does not elaborate on how music is chosen or created for “Music & Sound Design” element.

## Discussion and conclusion

With the continuous evolution of the VR market, VR devices are experiencing growing adoption within households. Thus, VR rehabilitation emerges as a valuable avenue, enabling patients to undergo rehabilitation treatment at home using VR devices. This substantially reduces the human and economic burden. Despite the existing inadequacy in clinical research evidence, it is apparent that VR rehabilitation holds tremendous potential [69]. VR rehabilitation serious games can offer patients an enhanced rehabilitation experience [27]. Consequently, driven by the pressing demand for rehabilitation and the evolving landscape of the VR market, VR rehabilitation has progressively emerged as a prominent research topic.

Despite the incorporation of game elements by many scholars to augment the enjoyment of rehabilitation training, there remains a deficiency in in-depth research on the design methods of VR rehabilitation serious games [17]. VR rehabilitation serious game deviates from commercial counterparts, necessitating meticulous consideration of clinical rehabilitation requirements alongside the physiological and psychological conditions of the patient population. Distinguishing from traditional rehabilitation training, VR rehabilitation serious games demand the integration of virtual reality technology. This integration encompasses the mapping of patient movements, the creation of virtual environments, and the amalgamation of game design theory to heighten patient training interests.

Therefore, this paper addresses the challenge that the MDA design framework tends to prioritize mechanisms in game design, which is not suitable for VR rehabilitation serious games. This paper commences with the functional decomposition of both rehabilitation and games and considers the imperative of maintaining the long-term enjoyment of rehabilitation training for patients, coupled with the hardware limitations of HMD, a VR rehabilitation serious game design framework named CFI is proposed. However, there are still some aspects remain to be discussed.

### The Player/Game/Therapy Model

The Player/Game/Therapy Model, proposed by Made et al.[70], provides a framework for analyzing therapeutic games by considering three key aspects: player, game, and therapy. Concerning *Therapy*, the model delves into treatment goals and anticipated outcomes. Regarding *Player*, the model investigates demographic information and the health status of the target player group to ensure that the game aligns with the characteristics and requirements of the intended players. In terms of the *Game*, the model considers aspects such as challenges, variability, and user experience. It aims to create an engaging and enjoyable gaming experience while also providing appropriate levels of challenge and adaptability to cater to different skill levels and stages of rehabilitation.

The proposed framework also underscores the importance of incorporating health status and demographic information into the design process. Within the “Clinical” part, gathering health information of the target population, such as conducting physical function assessments and evaluating patient status, is deemed necessary. Similarly, when crafting an engaging narrative in the “Interesting” part, patient characteristics should be considered. For instance, plotlines should be tailored to suit the preferences of different patient characteristics. In VR content, where immersion is heightened, designing stories that align with patient characteristics becomes even more critical. Furthermore, considering that VR technology is still relatively novel, the pace of patient acceptance should be considered. Introducing familiar processes, like starting with low difficulty settings in the “Difficulty” element, can help patients acclimate to the virtual environment. Additionally, it’s crucial to recognize the varying susceptibility of different demographic groups to VR-related side effects, such as motion sickness and visual fatigue, during the design phase.

The proposed framework significant emphasis on game challenge, variability, and user experience within the “Function” and “Interesting” part. It seeks to enhance game challenge and variability by incorporating elements of uncertainty into “game rules” and adjust difficulty levels dynamically. Additionally, the framework utilizes a “reward cycle” within the “Interesting” part to imbue scoring with meaning and employs external motivators to foster internal motivation, such as a sense of control and achievement, thus promoting long-term engagement.

Within this framework, the *Therapy* is primarily concentrated in the “Clinical” part. Here, clarify the rehabilitation goals of the patient through “Stage goals”. Elucidate the training methods and seamlessly integrate this content into the game within “Rehab Motion Features”.

### People, Aesthetics, Context, and Technology (PACT)

PACT, proposed by Charles et al. [71], is a participatory design framework for the gamification of rehabilitation systems. This framework is structured into three stages, wherein the dimensions of *People*, *Aesthetics*, *Technology*, and *Context* are reflected to varying degrees.

The proposed framework can be categorized into three parts: “Clinical,” “Function,” and “Interesting.” Within the “Clinical”, the primary dimensions reflected are *People* and *Technology*. Specifically, *People* pertains to clinical information regarding the patient population, while *Technology* involves the extraction of training motion features from patients in “Rehab Motion Features”.

Within the “Function”, all the four dimensions are reflected. Regarding *People*, it is reflected in “Duration” (visual fatigue) and “Feedback” (a sense of achievement by positive feedback). In terms of *Technology*, VR technology is utilized in motion capture and mapping, feedback triggering mechanisms, and DDA. *Aesthetics* involves designing rehabilitation training movements as a fun goal. *Context*: within “movement mapping”, the sufficient exercise space is ensured for safety.

In the “Interesting” part, the primary reflection is *People*, *Technology*, and *Aesthetics*. Regarding *People*, within “story”, patient characteristics are considered. In terms of *Aesthetics*, the focus is on enhancing the visual and auditory elements of the game to elevate the story atmosphere and increase immersion. For *Technology*, utilizing LLM large models can assist in designing the story plot. Additionally, considering that delays in VR can lead to motion sickness, the solution should be made to minimize GPU rendering and CPU computing consumption to reduce latency.

In the VR context, the dimensions of *People*, *Technology*, *Aesthetics*, and *Context* necessitate additional considerations. Under *People*, it is crucial to account for the varying sensitivity of individuals to the side effects of VR, ensuring tailored experiences for different patients. Within *Technology*, VR technology predominantly focuses on human–computer interaction, encompassing aspects such as motion capture and realistic modeling. In terms of *Aesthetics*, further research is warranted on how human perception, including concepts of agency and ownership, evolves within the virtual environment. This perception profoundly influences the patient’s experience and engagement in the virtual setting. Regarding *Context*, ensuring patient safety during virtual environment training across diverse settings remains a paramount concern, demanding meticulous attention to environmental factors and potential hazards.

### Serious Game Design Assessment Framework (SGDA)

SGDA is an assessment framework proposed by Mitgutsch and Alvarado in 2012 to assess the design quality and impact of serious games on players [72]. This framework divides games into six elements: (1) Purpose, (2) Content, (3) Mechanisms, (4) Fiction & Narrative, (5) Aesthetics & Graphics, and (6) Framing. The assessment of this serious game involves assessing how these elements interrelate and collectively support the overall purpose of the game. There are mapping relationships between the 6 elements of the SGDA and some elements within proposed framework, as outlined in Table 3.

**Table 3:** the mapping relationships between the 6 elements of the SGDA and proposed framework

SGDA	CFI
Purpose	Stage goal
Content	Feedback, Motion Mapping
Mechanics	Game rules, Feedback, Reward Mechanism, Difficulty
Fiction & Narrative	Story
Aesthetics & Graphics	Art Design, Music & Sound Design
Framing	Game rules, Difficulty, Feedback, story

Moreover, within the VR context, it is crucial to assess potential side effects of the game, such as motion sickness and visual fatigue, as these factors significantly impact the patient’s gaming experience.

However, a limitation of this paper is that patient recruitment for experiments has not been initiated, primarily due to the absence of an established evaluation method for HMD-VR rehabilitation serious games. The widely utilized game evaluation framework is currently the GameFlow model, which predominantly focuses on the entertainment aspect of traditional electronic games [73]. As the gaming landscape evolves, various scholars have introduced specific GameFlow evaluation models tailored to the characteristics of different games [74, 75]. Nonetheless, HMD-VR rehabilitation serious games possess their unique attributes. Firstly, they intend to assist patients in rehabilitation, necessitating a close integration with clinical practice. Thus, evaluating the extent of integration between games and rehabilitation becomes a pivotal challenge. Secondly, employing HMD as a gaming medium requires careful consideration of feedback, control, and immersion [76] Therefore, our future work will concentrate on proposing an evaluation method tailored to VR rehabilitation serious games and initiating patient recruitment for experimental studies.

This paper offers a systematic theoretical framework guiding the design and development of VR rehabilitation serious games, providing viable methodologies for integrating virtual reality technology into future rehabilitation treatments. We aspire that this research will catalyze innovative endeavors, contribute to the continued advancement of VR rehabilitation technology, and pave the way for more personalized and efficient rehabilitation modalities for patients.

## Data Availability

No datasets were generated or analysed during the current study.

## Code availability

The code of the designed game is available from the corresponding author on reasonable request.
